# Cancer Cell Invasion Is Enhanced by Applied Mechanical Stimulation

**DOI:** 10.1371/journal.pone.0017277

**Published:** 2011-02-17

**Authors:** Shalini Menon, Karen A. Beningo

**Affiliations:** Department of Biological Sciences, Wayne State University, Detroit, Michigan, United States of America; University of Bergen, Norway

## Abstract

Metastatic cells migrate from the site of the primary tumor, through the stroma, into the blood and lymphatic vessels, finally colonizing various other tissues to form secondary tumors. Numerous studies have been done to identify the stimuli that drive the metastatic cascade. This has led to the identification of multiple biochemical signals that promote metastasis. However, information on the role of mechanical factors in cancer metastasis has been limited to the affect of compliance. Interestingly, the tumor microenvironment is rich in many cell types including highly contractile cells that are responsible for extensive remodeling and production of the dense extracellular matrix surrounding the cancerous tissue. We hypothesize that the mechanical forces produced by remodeling activities of cells in the tumor microenvironment contribute to the invasion efficiency of metastatic cells. We have discovered a significant difference in the extent of invasion in mechanically stimulated verses non-stimulated cell culture environments. Furthermore, this mechanically enhanced invasion is dependent upon substrate protein composition, and influenced by topography. Finally, we have found that the protein cofilin is needed to sense the mechanical stimuli that enhances invasion. We conclude that other types of mechanical signals in the tumor microenvironment, besides the rigidity, can enhance the invasive abilities of cancer cells *in vitro*. We further propose that *in vivo,* non-cancerous cells located within the tumor micro-environment may be capable of providing the necessary mechanical stimulus during the remodeling of the extracellular matrix surrounding the tumor.

## Introduction

The defining moment in the classification of a tumor as benign or malignant lies in the tumor cells ability to breach the basement membrane. The extension of invasive structures, such as invadopodia, allows the tumor cell to penetrate the basement membrane and interstitial stroma through enzymatic and physical means [1]–[3]. However, the tumor cell will not go far without the additional ability to migrate. The tumor cells acquisition of invasive and migratory properties provide the means to enter and exit the lymphatic or the vascular system and establish secondary tumors in foreign tissue, thereby completing the complex sequence of events within the invasion-metastasis cascade [4], [5]. It is these secondary tumors that account for greater than 90% of cancer deaths, yet our understanding of invasion and metastasis is incomplete. Much of the research has focused on intrinsic genetic and biochemical factors that trigger primary tumor formation and subsequent metastasis. However, more recent studies have identified both physical and biochemical factors within the tumor microenvironment that also contribute to cancer progression [6], [7].

The stroma surrounding a tumor is continually changing in composition and structure as the primary tumor cells progress to invasion and metastasis, a process termed stromagenesis [8], [9]. The tumor stroma becomes enriched in extracellular matrix (ECM) proteins and non-tumor cells including fibroblasts, macrophages, adipocytes, and pericytes [8]–[12]. Biochemical signaling from the stroma to the tumor cells can promote proliferation and invasiveness. For instance, tumor-associated macrophages establish an EGF-CSF-1 paracrine signaling loop with the tumor cells that promote tumor cell movement [10]. The mechanical properties of the stroma can also enhance tumor progression. For example, the stroma surrounding a tumor is enriched in both type I collagen and fibronectin, creating a denser and mechanically rigid tissue compared to normal tissue [7]. This increased rigidity enhances tumor cell proliferation and dissemination [13]–[15]. Recent studies also indicate that physically stretching fibronectin can trigger a mechanical response pathway in normal fibroblasts [16]–[18]. Given the increased amount of fibronectin in the stroma, these observations could suggest a potential mechanism for the mechanical response of tumor cells.

There are a number of mechanical forces, aside from the change in compliance, that may impact the progression of cancer. One such force could be derived from stromal cell movements or the matrix remodeling activity of the highly contractile cells of the stroma, including fibroblasts and myofibroblasts. Myofibroblasts have been shown to differentiate from normal tissue fibroblasts, and their production and remodeling of the ECM enhances proliferation and dissemination of the tumor cells [19], [20]. The accumulation of stromal myofibroblasts are a defining feature of the desmoplasia most commonly associated with invasive cancers of the breast, gastrointestinal tracts, lungs, pancreas, and squamous cell carcinomas to name a few [9]. In addition to the high level of type I collagen production, myofibroblasts are identified by their expression of alpha-smooth muscle actin [7], [9], [21], [22]. The alpha-smooth muscle actin associates with non-muscle myosin to form highly contractile microfilamentous units that terminate at the surface of a myofibroblast in a fibronexus [23]. These are characteristic features of myofibroblasts and form a mechano-transduction system that functions in inside-out and outside-in force transmission [23]–[25]. In remodeling the ECM within the stroma, the myofibroblasts produce a mechanical stimulus as they tug and pull on the fibers [26]. This leads us to the question we address in this study. Could the applied mechanical forces generated by the remodeling of the ECM and pulling on the ECM by stromal cells contribute to the invasive properties of a tumor cell? Can they provide a “come hither” stimulus that encourages the tumor cells to leave the tumor?

Here we report that a mechanical stimulus of pulling and releasing applied to a collagen matrix *in vitro* does indeed enhance the invasion of cancer cells in a fibronectin dependent manner. This ability appears to be unique to cancer cells that are known to be highly invasive, as poorly invasive and normal cells do not respond in the same way to this stimulus. Finally, using gene silencing we determined that cofilin, a normal component of invadopodia, is required to sense this mechanical signal for enhanced invasion. This study suggests that physical factors, beyond compliance, are involved in promoting existing invasive behavior in cancer cells and that mechanical signals transmitted from the physical activity of cells within the stroma may potentiate cancer progression.

## Materials and Methods

### Cell Culture

HT1080 human fibrosarcoma cells, B16F10 mouse melanoma cells and mouse embryonic fibroblasts (MEF) cells used in this study, were purchased from ATCC and are cultured and maintained in Dulbecco's Modified Eagle's Medium - high glucose (Sigma) and 10% FBS (Hyclone). Cells were passed by trypsinization using 0.25% Trypsin-EDTA, the reaction is terminated with complete media. The passage number of any cell type never exceeds eight passages.

### Invasion Matrices

To create a culture well for thick (1 mm) matrices, an activated coverslip [27] was attached with vacuum grease to the bottom of a culture dish (Nunclon) into which a 20 mm hole had been drilled.

The matrix was composed of 2.5 mg/ml (or 4.5 mg/ml, Figure S2) type I collagen (PureColl and Nutragen, Advanced Biomatrix), 20 µg/ml fibronectin (Sigma) and 4 µl of 1–2 µm carboxylated paramagnetic beads (Polysciences Inc.). The pH of the mixture was adjusted to 7.4±0.2 with 0.1 N NaOH and 10X PBS. For “Collagen only” substrates, everything except fibronectin is added to the matrix mix. All the components were chilled and mixed at 4°C. 500 µl of the matrix solution was added to a chilled culture well, and a 25 mm coverslip was dropped onto the gel mixture to obtain a flat surface. For polymerization, the matrix solution was placed at 37°C for 30 minutes. Following polymerization, 3 ml of media was added to the substrates and the top coverslip was removed. The substrates were then sterilized in a culture hood under ultraviolet light for 15 minutes at a distance of 25 inches from the light source.

### Invasion Assay

Cells were seeded at 1.5×10^4^ cells/ml onto sterilized matrix and allowed to adhere for 1 hour at 37°C/5% CO_2_. For each experiment, one seeded matrix was incubated at 37°C/5% CO_2_ 1.5 cm above a rare earth magnet of 12,100 Gauss (25 mm in diameter and 5.5 mm in thickness). A second seeded matrix was incubated outside the magnetic field. The magnet was rotated below the culture at 160 rpm (2.6 Hz) in an orbital field of 2 cm on an orbital shaker (Barnstead Thermolyne, Roto Mix-Type 50800). This rotation frequency was maintained the same for all assays described. The invasion assay was also performed with the magnet rotated at lower frequencies (8 and 90 rpm (0.13 and 1.5 Hz)) as indicated. The cellular response was recorded for 25 randomly selected microscope fields at 24 hours using a 10X phase objective on an Olympus IX81 Microscope. Cell counts were recorded at eight increments of 100 µm/step within the z-plane of the matrix. Percentage invasion was calculated as the percent of invaded cells in comparison to the total cell count. Statistical analysis was performed using the two-tailed students T-test.

The peptide inhibitor experiments were performed as above; 1.5×10^4^ cells/ml were seeded onto the substrates followed by 100 µg/ml of GRGDS peptide or GRGES (control) peptide (Bachem Americas Inc.) suspended in water. Percent invasion was calculated 24 hours after the start of stimulation.

### Upward Invasion Assay

Culture wells without the substrates were prepared as described above. However, cells were first seeded directly onto the glass coverslip coated with a thin layer of type I collagen (200 µg/ml) and fibronectin (62.5 µg/ml) before overlay of the matrix. The cells were allowed to adhere overnight in media at 37°C and 5% CO_2_. The media was removed and cells were then overlaid with the unpolymerized collagen/fibronectin matrix as described above. Media was replaced following polymerization. For each experiment, one seeded overlaid matrix was cultured 1.5 cm below a rare earth magnet of 12,100 Gauss (25 mm in diameter and 5.5 mm in thickness) and a second was maintained outside the magnetic field. The magnet was rotated above the culture held in a stand placed on the orbital shaker (Barnstead Thermolyne, Roto Mix-Type 50800) and rotated at 160 rpm (2.6 Hz) in an orbital field of 2 cm. Percent invasion and statistical analysis were described above.

### Actin Depolymerization

HT1080 cells were seeded onto collagen/fibronectin substrates. After the cells had adhered and spread on the substrates, 2 µM of Cytochalasin B (Sigma) resuspended in DMSO or a corresponding volume of DMSO was added to separate plates. These were then directly used for invasion assay.

### Cofilin Knockdown

CFL1 siGENOME SMARTpool and Off-target siRNA (Dharmacon RNAi Technology, Thermo Scientific) were used to silence the expression of Cofilin and as controls, respectively. RNA's were introduced into cells by nucleofection using an Amaxa Nucleofector II and solutions from Kit T. Proteins were extracted for western analysis from silenced and control HT1080 cells using a triple detergent lysis buffer (100 mM Tris-Cl, 300 mM NaCl, 0.5% sodium deoxycholate, 0.2% SDS, 2% Nonidet P 40) containing Protease Inhibitor Cocktail (Sigma) at 24, 48 hours and 72 hours post nucleofection to confirm knockdown. Anti-cofilin monoclonal antibody, ab54532 (Abcam) and anti-mouse HRP-labeled antibody (Amersham) were used to probe the western blots and detected with ECL Plus Western Blotting Detection Reagents (Amersham).

### Invasion Assay Using Cofilin siRNA and Cytochalasin B Treated HT1080 Cells

Invasion assay was performed using Control siRNA and Cofilin siRNA treated HT1080 cells. Since cofilin knockdown is efficient 48 hours post nucleofection, the treated cells were seeded onto the substrates at the 48 hour time point. After the cells had adhered, one seeded matrix for each of the conditions was placed above the magnet rotating at 160 rpm (2.6 Hz), whereas the other was placed outside the magnetic field. The assay was also performed using Cytochalasin B or DMSO treated cells. In each case, one seeded matrix was provided magnetic stimulation at 160 rpm (2.6 Hz) whereas the other matrix was placed outside the magnetic field. The cellular response for each of the four conditions was measured 24 and 48 hours after the start of stimulation. Percentage invasion was calculated and statistical analysis was performed using a two-tailed student T-test.

### Western Blot of Fibronectin Secretion by HT1080 Cells

1.5×10^4^ cells/ml HT1080 cells were grown in serum free DMEM medium and seeded onto collagen-only matrices, prepared as described above, and the standard invasion assay was performed. After 24 hours of stimulation, the cultures were scraped into a microfuge tube containing 2 mg/ml of Collagenase Type 4 (Worthington Biochemical Corporation) in Hanks' Balanced Salt Solution (Gibco, Invitrogen). The collagen matrix was solubilized for 10 minutes by gently shaking the tube at 37°C and cells were pelleted by centrifugation at 2000 rpm for 5 min, the supernatent was used for analysis. Cell extracts of HT1080 cells and MEF cells cultured on 100 mm polystryrene culture dishes to 80% confluency over 48 hours were also prepared. The cell lysis and protein extraction were performed as described above. Standard SDS-PAGE was performed using 30 µg of total protein from MEF and HT1080 cell extracts and 35 µl of collagenase suspension. Western blots were prepared and probed with mouse monoclonal [IST-9] to fibronectin (1∶300), ab6328 (Abcam) in 5% milk in TBS followed by a HRP Goat Anti-mouse Ig (BD Pharmingen) secondary antibody (1∶1000) and detected as above.

## Results

### Structural Design of the Mechanical Invasion Assay

**Figure 1:**
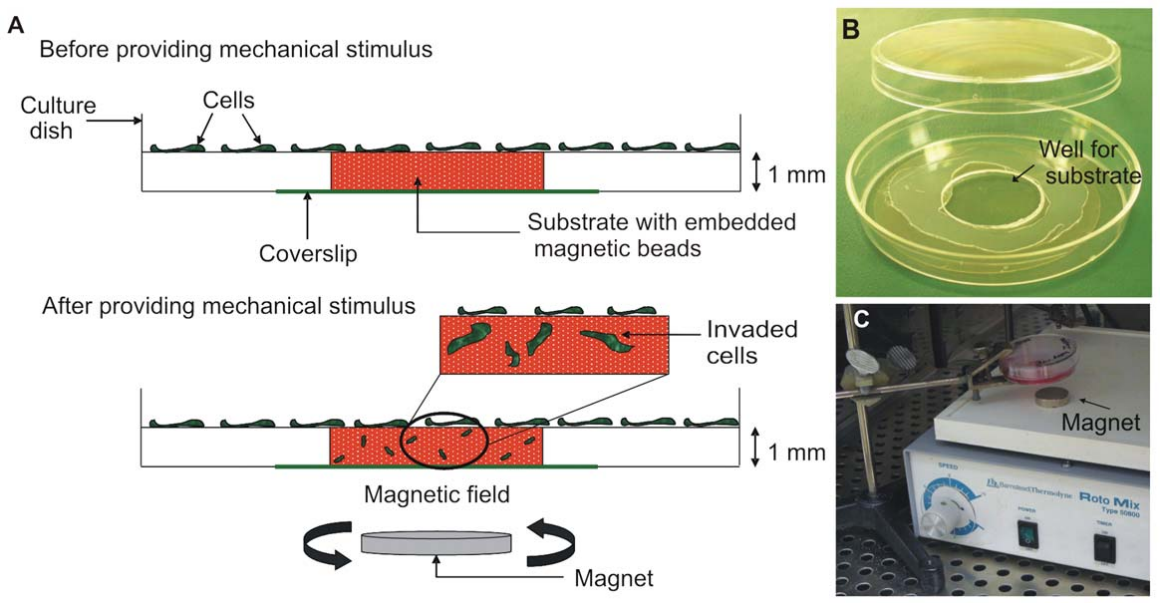
The mechanically enhanced invasion assay. **A**) A well is created in a 60 mm culture dish and filled with a type I collagen/fibronectin matrix containing 2 µm paramagnetic beads. Cells are seeded onto the surface of the matrix and either cultured outside of a magnetic field or cultured 1.5 cm above a rotating rare earth magnet. Upon stimulation, cells invade the matrix. **B**) 60 mm plate with a 20 mm hole drilled into it, with an activated coverslip glued to the bottom, creates a well for the matrix. **C**) The culture is suspended 1.5 cm above a rare earth magnet placed on an orbital shaker within a typical cell culture incubator. See the methods section for details.

The goal of this study was to determine if applied mechanical stimulation, such as those simulating the re-modeling of the extracellular matrix, could enhance the process of invasion. To address our hypothesis, we designed a new assay system where mechanical stimulation could be applied in the absence of secreted biochemical factors. Our intention was to create an assay that used commercially available components, required standard equipment, provided control of biochemical and mechanical parameters, all in a framework that was optically compatible with an ordinary fluorescent microscope. We chose to use a type I collagen matrix commonly used for invasion assays, reasoning that the stroma is highly enriched in this extracellular matrix protein. Carboxylated fluorescent paramagnetic micro-beads were embedded within the matrix to provide mechanical stimulation. To produce a transient magnetic pull, without the need for a micron size electro-magnet, we rotated a rare earth magnet on a rotating mixer beneath the culture while the culture was suspended above the magnet (Figure 1A). The entire culture system can be maintained within a standard tissue culture incubator (Figure 1B, C).

**Figure 2:**
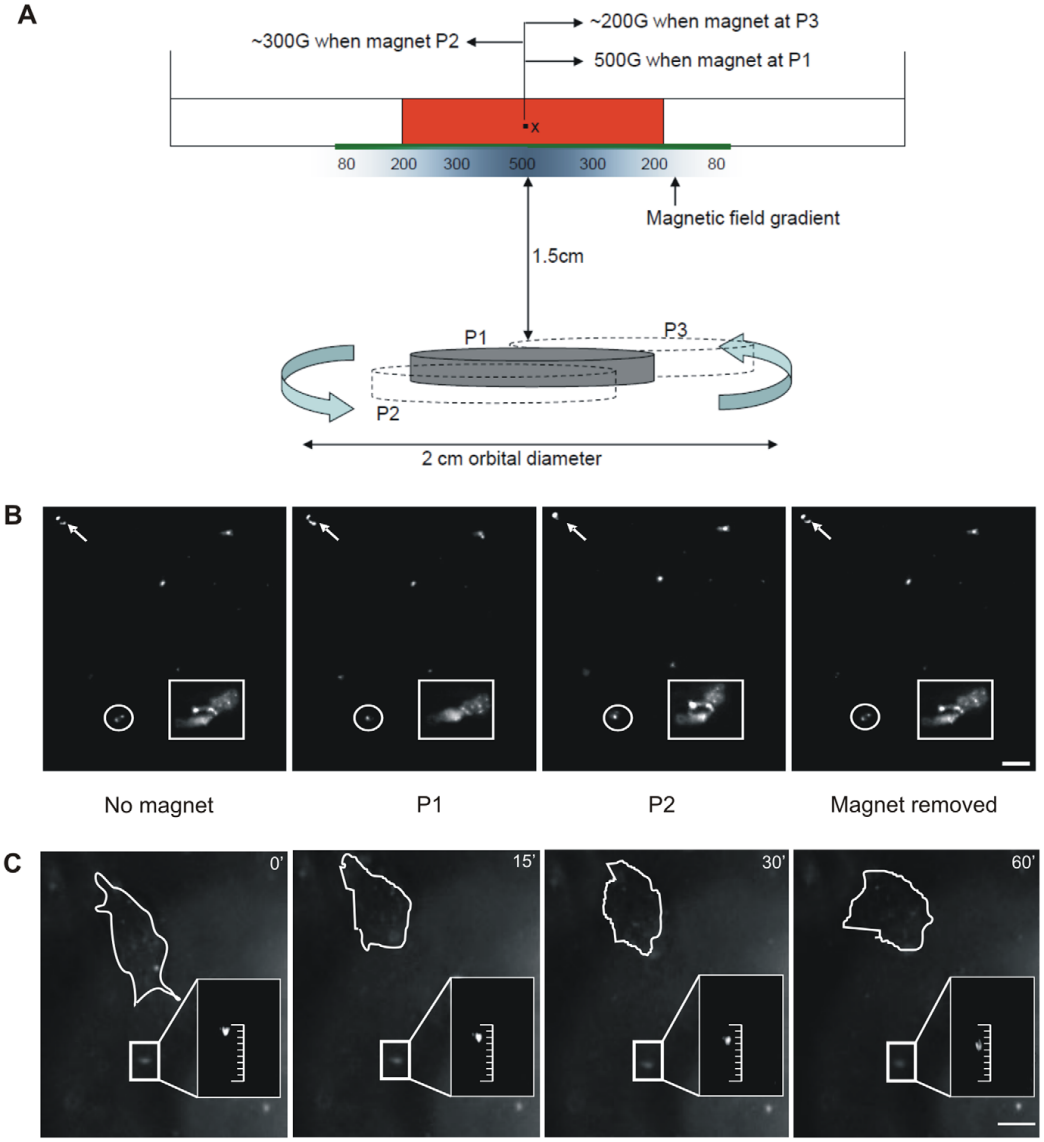
Stimulation of paramagnetic beads. **A**) A rare earth magnet placed 1.5 cm below a matrix produces a gradient field ranging from 500G to 80G within the matrix as it rotates in a 2 cm orbit. A paramagnetic bead at position X would receive a magnetic force of 500G, ∼300G and ∼200G when the magnet is orbiting at positions P1, P2 and P3 respectively. **B**) Series of four images depicting the displacement of beads by the magnet when held in stationary positions within the orbit. Clusters of beads responding to the mechanical stimulus and showing a positional shift have been demarcated using a circle, a square and an arrow. From left to right, image one is outside the magnetic field while the second and third images were taken with the magnet held in positions P1 and P2 respectively. The final image demonstrates the beads return to their original position after the magnet is removed. **C**) MEF cellular extensions cause fluorescent bead displacement. Four images (0, 15, 30 and 60 minutes) from a single focal plane were selected from a series of 30 phase images taken every 2 minutes of a MEF cell within a collagen/fibronectin matrix. Cell outlines and corresponding fluorescent bead images are shown. A bead undergoing displacement is outlined using a white rectangular box. The area within the box from all four images has been enlarged and displayed with an inset ruler to show the bead displacement more clearly. The contrast of the magnified images have been altered to better reflect the position of the bead in each case. Mag. Bar  = 10 µm.

To verify that the magnet was capable of producing enough magnetic force and that the embedded beads responded to the force in a transient manner, we used a magnometer to measure the magnetic force at defined experimental distances. We discovered a magnetic bead at a fixed point within the center of the culture could be subjected to a range of 500 to 80 Gauss as the rare earth magnet rotates 1.5 cm beneath the culture dish while completing an orbit of 2 cm at 160 rpm (2.6 Hz) (Figure 2A). Simulation at these distances under the microscope resulted in bead displacements of approximately 0.5–5 µm (Figure 2B, Movie S1, Movie S2). Beads were observed to spring back to their original position in the x-y plane after the magnet was removed, indicative of their attachment to the collagen matrix and maintenance of the integrity of the gel network. To determine the physiological significance of this displacement, we recognized that we could calculate the amount of force that was applied on the bead by the magnet, however a more tangible test would be to observe MEF cells extending and retracting extensions within our controlled culture system. We recorded bead displacements in the x-y plane from cellular extensions of MEF cells that range from 0.08–5.1 µm (Figure 2C, Movie S3). This is a conservative comparison to the types of displacements that could occur in the stroma given that the most contractile cell type found there, the myofibroblasts, produce considerably more force than a MEF [28], [29].

### Mechanical Stimulation Enhances the Invasion of Cancer Cells

Invasive structures have previously been described in both inherently normal invasive cells and in those that have acquired their invasive capacity during cancer progression [30]. We reasoned that it was unlikely that mechanical stimulation would induce a previously non-invasive cell type to invade and hence we tested cells known to be highly invasive in our assay system. We chose to test the human fibrosarcoma cell line HT1080 and the mouse melanoma cell line B16F10, whereas the non-invasive MEF cell line served as control.

**Figure 3:**
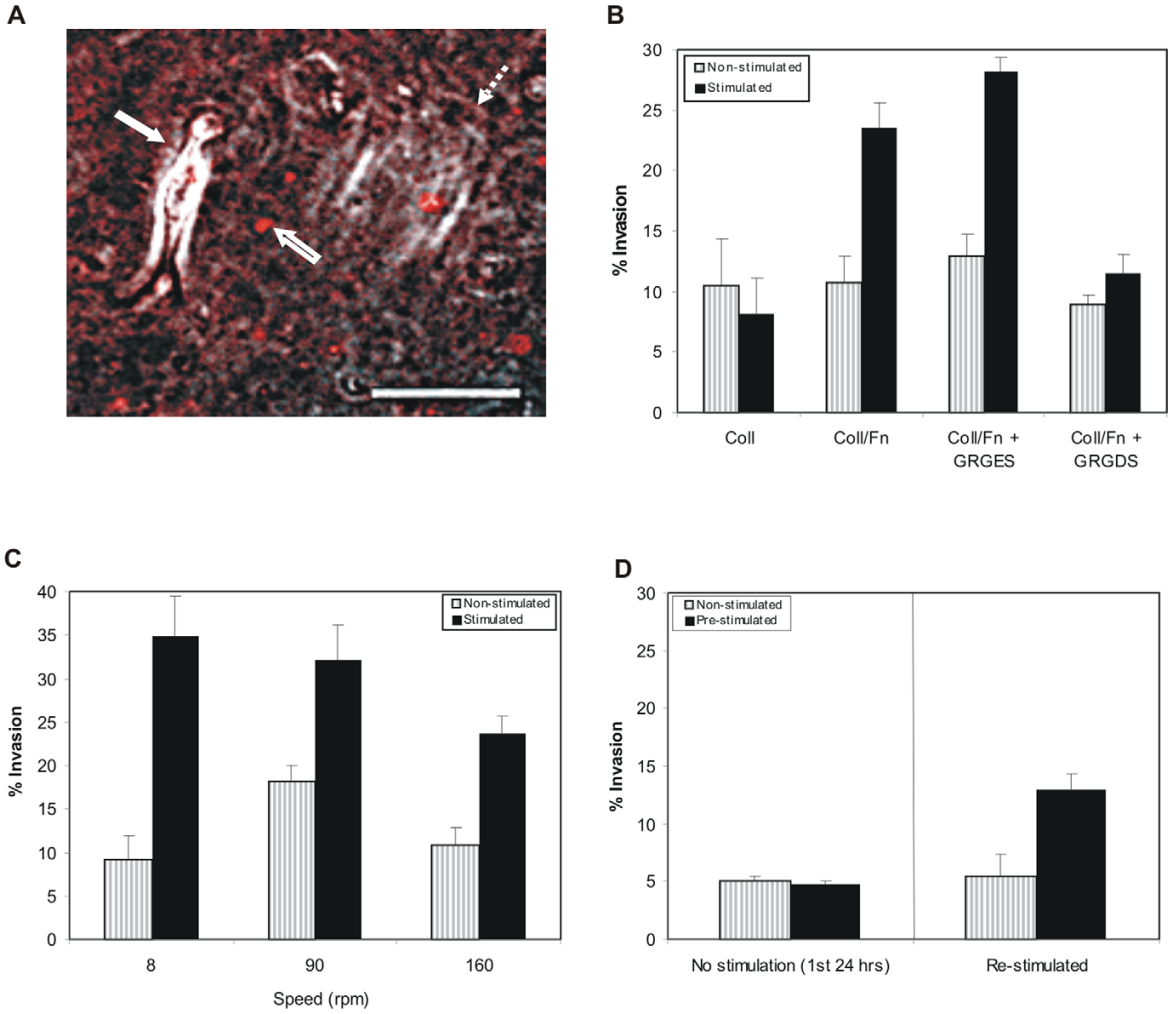
Enhanced invasion of mechanically stimulated cultures of cancer cells. **A**) HT1080 fibrosarcoma cells were seeded onto type I collagen/fibronectin matrices containing paramagnetic beads and cultured either under magnetic stimulation or without stimulation. A combined phase and fluorescent image of a mechanically stimulated culture were superimposed. The solid arrow points to a cell that has invaded. The dotted arrow indicates a second cell within another focal plane. The empty arrow points to a fluorescent paramagnetic bead. Mag. Bar  = 50 µm. **B**) Invasion of HT1080 cells under mechanically stimulated and non-stimulated conditions was performed in matrices containing either type I collagen (2.5 mg/ml) or both type I collagen and fibronectin, or collagen/fibronectin in the presence or absence of RGD peptide. 25 fields of cells were counted 24 hours after seeding at multiple depths within each matrix beginning 5 µm below the surface of the matrix and progressing towards the farthest depth of 800 µm. The percent of invading cells was 2-fold higher in stimulated cultures when compared to controls (*P*<0.05) in matrices containing both ECM proteins. Similar results were obtained when the control peptide GRGES was added to the media. The percent invasion was approximately the same with or without stimulation when fibronectin was absent. Addition of the GRGDS peptide also resulted in inhibition of enhanced invasion upon mechanical stimulation. **C**) A 20-fold difference in the frequency of stimulation does not influence the percent of cell invasion. The percent of invading cells 24 hours after stimulation at magnetic rotation speeds of 8, 90 and 160 rpm (0.13, 1.5 and 2.6 Hz). An insignificant difference was found between cells stimulated at 8 and 160 rpm (*P*>0.05). Data represents three independent experiments, of 25 fields. **D**) Type I collagen/fibronectin matrices containing paramagnetc beads were pre-stimulated for 24 hours. These matrices were then seeded with HT1080 cells and counted 24 hours after seeding, during which period both the pre-stimulated and the control plates were not stimulated (left panel). These cultures were then either continued or placed over the magnet (right panel), data obtained 24 hours after stimulation. Data represents two independent assays of 15 fields of cells at a depth range of 800 µm. Two-tailed analysis using student t-test.

These cell types were tested individually for their ability to respond to the mechanical stimulation provided in the assay. In brief, cells were seeded onto prepared matrices, as described in methods, and allowed to adhere for 30 minutes before beginning the stimulation. Cells cultured on matrices of identical composition, but not subjected to magnetic stimulation, served as controls. Cells cultured on matrices lacking magnetic beads, but subjected to magnetic stimulation served as additional controls. Invasion was observed under the microscope beginning at 5 µm from the surface to a depth of 800 µm within the matrix (Figure 3A). The number of invading and non-invading cells were counted after 24 hours of stimulation and calculated as the percent invasion.

We initially seeded our cells onto matrices comprised only of type I collagen. Upon stimulation we did not observe enhanced invasion (varying between 5 and 10% invasion in stimulated and non-stimulated cultures). However, not only is type I collagen abundant in the stroma, but the collagen binding ECM protein fibronectin is also enriched [7], [31]. Thus, we compared matrices composed of collagen alone to those of both collagen and fibronectin, with and without stimulation. Under these conditions we observed a significant difference in the number of invading cells in mechanically stimulated verses non-stimulated culture environments for the invasive cell types when collagen/fibronectin matrices were used (Figure 3B). A two-fold increase in the percentage of invading cells in the stimulated (23%) as compared to the non-stimulated matrix (10%) was consistently observed in these cultures (P<0.05). These results indicated that an applied stimulus was capable of enhancing invasion of cancer cells, but required in the presence of fibronectin for the mechanical response. Furthermore, we found that non-invasive MEF cells failed to invade both in the presence or absence of mechanical stimulation into collagen/fibronectin matrices, suggesting the need for a cell to have a pre-defined ability for invasion.

To confirm the importance of fibronectin for the mechanical response we inhibited cell-fibronectin interactions with RGD inhibitory peptides. Cells were treated with the GRGDS peptide or a control GRGES peptide after seeding onto the collagen/fibronectin matrices. The percent invasion was normal in the presence of the control GRGES peptide (28% with stimulation and 13% without stimulation) while mechanically stimulated invasion was inhibited by the RGD peptide (9% with stimulation and 11.5% without stimulation, P>0.05). These results not only support the fact that fibronectin is necessary for the mechanically stimulated invasion, but suggest the “basal” level of ∼10% invasion observed in collagen/fibronectin (non-stimulated) and collagen (stimulated and non-stimulated) cultures is fibronectin independent. In addition, these results suggest that any fibronectin secreted by the HT1080 cells into the matrices (although undetectable by western blot; Figure S1) has little affect on the mechanical response.

Due to the heterogeneity of cell types and cell numbers within the stroma it was unclear at what frequency the stimulus should be applied. To determine if the frequency of bead stimulation was a factor in enhanced invasion, we adjusted the speed of the rotating magnet, rotating at speeds of 8, 90 and 160 rpm or 0.13, 1.5, and 2.6 Hz, respectively. The percent of invasion did not differ significantly between the cultures stimulated at 8 and 160rpm (*P*>0.05; Figure 3C). These results demonstrated that, within a 20-fold range of frequency, enhanced invasion in response to mechanical stimulation is unaffected.

Invasive cells encounter physical barriers within the connective tissue or tumor stroma and are likely to follow the path of least resistance [32]. In addition, they are likely to invade along paths in which matrix associated soluble factors have been released [19], [33]–[36]. Based on this knowledge, it was important to ensure that neither of these factors contributed to the enhanced invasion observed in our assay.

One way in which our matrix could generate paths of least resistance for cell invasion would be through a permanent remodeling created by the movement of the embedded beads. To test this possibility, we pre-stimulated the matrices over the rotating magnet for 24 hours prior to seeding the cells. After 24 hours of culture on the pre-stimulated matrices, but outside of the magnetic field, we did not observe enhanced invasion (Figure 3D, left panel). In addition, the media of the pre-stimulated matrix was not changed prior to seeding the cells. This eliminated the potential that soluble factors in the matrix were being released by the tugging of the beads on the matrix and contributing to the enhanced invasion. However, when these same cell cultures grown on the pre-stimulated matrix were then given magnetic stimulation, enhanced invasion was again observed (Figure 3D, right panel). Taken together, these results suggest that any remodeling or release of soluble factors from the matrix due to the movement of the magnetic beads does not contribute to the enhanced invasion we observe upon mechanical stimulation.

### The Invasion Response is Enhanced whether the Stimulus is Delivered from Top or Bottom

**Figure 4:**
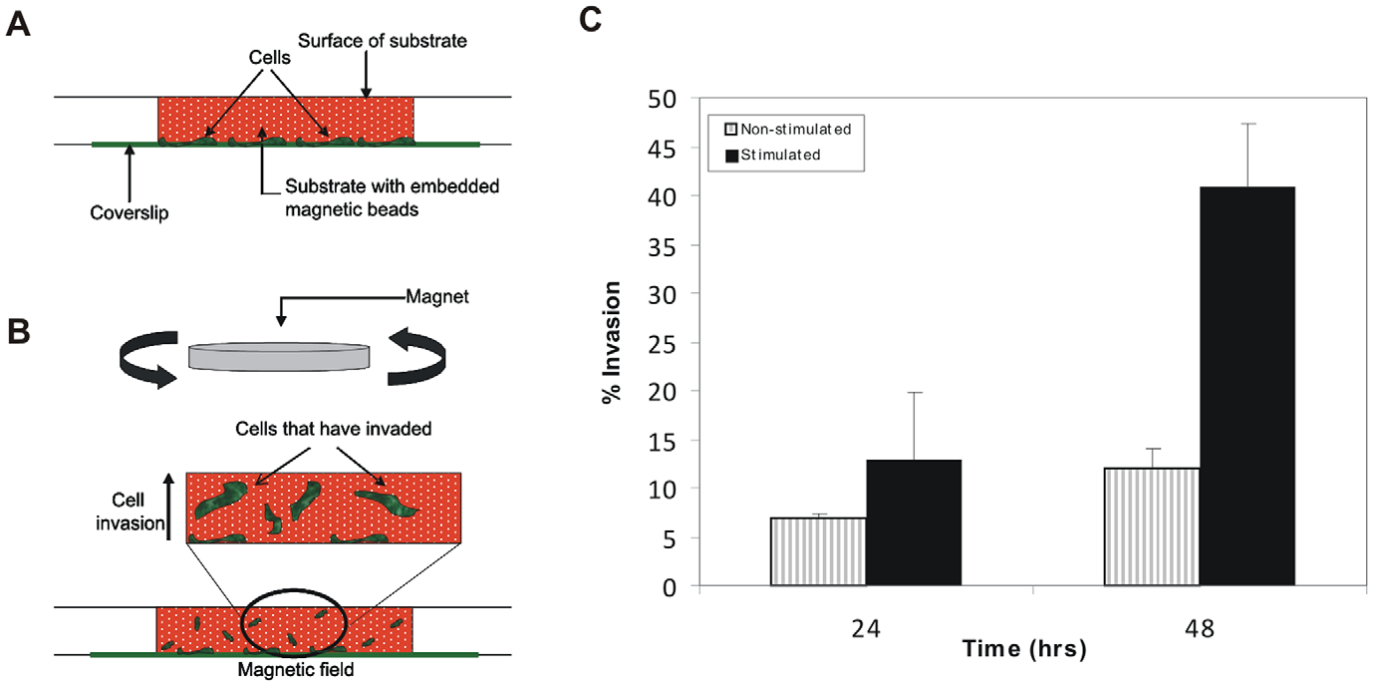
Upward Invasion Assay. **A**) HT1080 fibrosarcoma cells were seeded onto a collagen/fibronectin coated coverglass at the bottom of the well. After the cells had adhered, a type I collagen/fibronectin solution containing paramagnetic microbeads was overlaid onto the cells and allowed to polymerize. Cultures were either subjected to magnetic stimulation or grown outside the magnetic field. **B**) The magnet is rotated above the culture as cells start to invade up into the matrix. **C**) HT1080 cells seeded on a collagen-fibronectin coated coverslip and overlaid with a collagen/fibronectin matrix were cultured either in the presence or absence of a magnetic field. Percent invasion was calculated 24 and 48 hours following stimulation from three independent trials (15 fields were counted per culture). A difference in invasion (approx. 4–fold higher) between the stimulated cultures as compared to non-stimulated cultures was significant at 48 hours post-stimulation (*P*<0.005).

The dimensionality of the environment is known to influence cellular behavior. Specifically, HT1080 cells have been shown to change their migration speed and persistence in three dimensions [37]. In our initial experiments, the cells are seeded on top of the matrix, invading from the top downward, thus beginning in two-dimensions and moving into three. To address the influence of dimensionality on mechanical invasion we changed the orientation of the stimulus so the cells would invade upwards. To do this, we first seeded the cells onto collagen/fibronectin-coated coverslips before overlaying and polymerizing the collagen/fibronectin/magnetic bead solution over them (Figure 4A). The magnetic field was then applied to the top of the culture by rotating the magnet above the stationary culture (Figure 4B). After 24 hours of stimulation, we found the cells invaded just as well as they did when they were seeded on top of the matrices prior to stimulation (6% invasion in non-stimulated and 13% in stimulated cultures) (Figure 4B). However, we found by 48 hours the difference between non-stimulated invasion and stimulated invasion was even larger such that 12% of the cells invaded in non-stimulated versus 41% invasion in the stimulated cultures. Thus, an even greater enhancement of invasion occurs in the response to applied mechanical stimulation when the cells began in a three-dimensional environment.

### Cofilin and Actin are Required for Mechanically Stimulated Invasion

**Figure 5:**
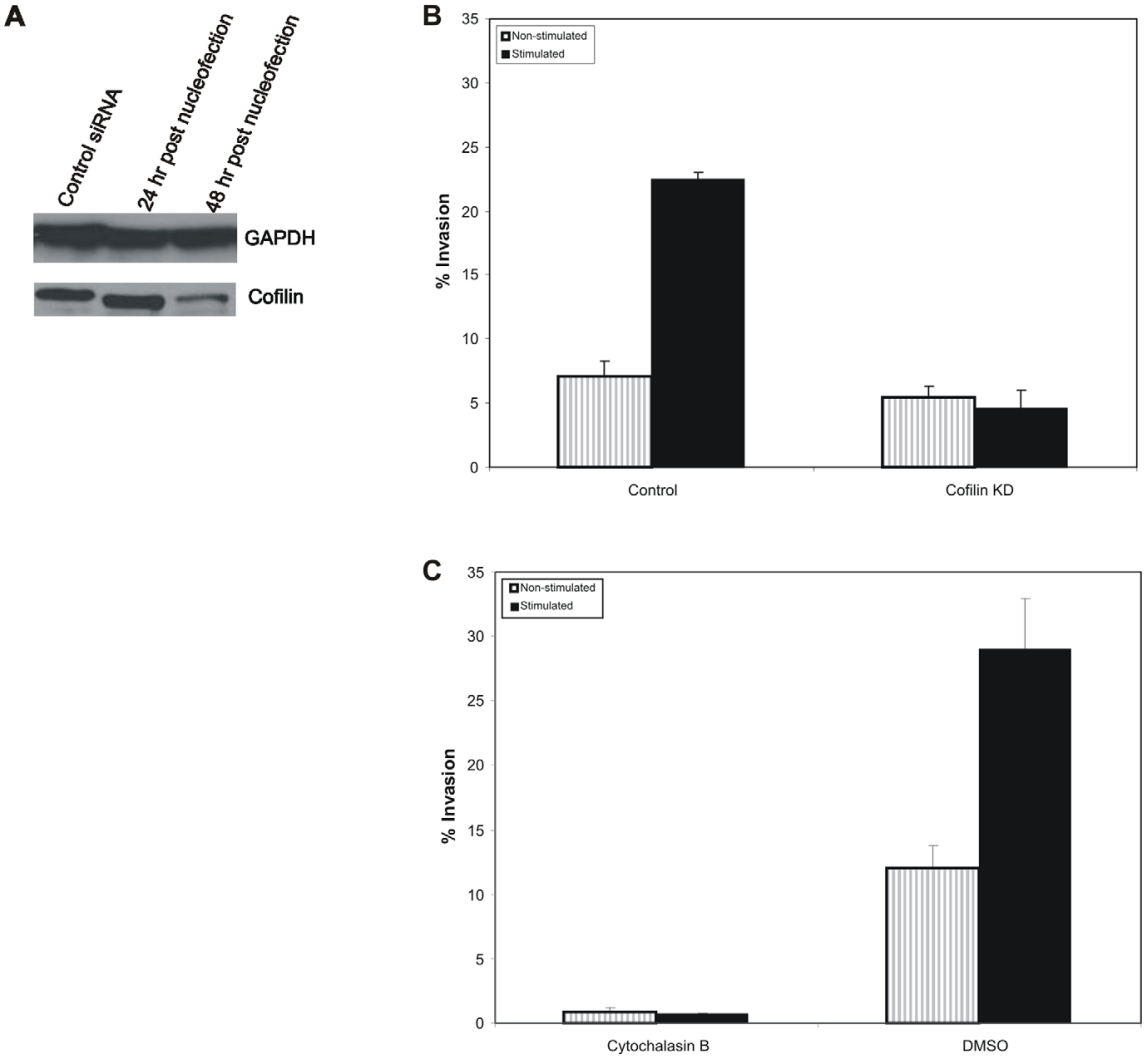
Silencing of Cofilin prevents mechanically stimulated invasion. **A**) Western blot of cofilin from lysates of HT1080 cells treated with off-target control siRNA (lane 1, from left), and cells cultured for 24 and 48 hours after nucleofection with cofilin siRNA (lanes 2 and 3 respectively). Cofilin expression is reduced 48 hours post-nucleofection. GAPDH was used as loading control. **B**) HT1080 cells nucleofected with control siRNA or Cofilin siRNA and cultured for 48 hours were seeded onto collagen/fibronectin matrices containing paramagnetic beads. The cells were cultured with or without stimulation for 48 hours and the percent of invading cells was calculated. Invasion assays using control siRNA treated cells were repeated twice (15 fields were counted per trial). Stimulated cells had 3-fold higher invasion as compared to non-stimulated cells (*P*>0.05). The assay using cells when cofilin was silenced, was repeated four times (15 fields were counted per trial). The percent invasion between stimulated or non-stimulated cultures was insignificant (*P*>0.05). **C**) HT1080 cells were seeded onto collagen/fibronectin matrices containing paramagnetic beads. Cells treated with 2 µM Cytochalasin B or DMSO were cultured with or without stimulation for 48 hours and the percent of invading cells was calculated. Data represents three independent assays.

A functioning actin cytoskeleton is required for the invasiveness of a number of tumor cells [38], [39]. To confirm the significance of actin dynamics in HT1080 invasion into type I collagen/fibronectin matrix, Cytochalasin B or control DMSO treated cells were tested in the invasion assay. As anticipated, both the mechanically stimulated and the non-stimulated invasion were inhibited. Less than 1% of the cells treated with Cytochalasin B invaded irrespective of whether they were mechanically stimulated (Figure 5C). In comparison, 12% of non-stimulated and 29% of stimulated, DMSO treated control cells invaded into the matrix (Figure 5C). As expected, invasion into a 3D matrix is dependent on the dynamics of the actin cytoskeleton.

Given that mechanical stimulation enhances an existing ability for invasion, it was important to identify other proteins that might sense the mechanical stimulation, but whose function is not dire to the formation of invasive structures as with actin. We tested the protein cofilin because it is vital for maturation of invadopodia, since reduced cofilin expression leads to the formation of less invasive invadopodia, but does not inhibit invasion [40]. Cofilin is also important in directional sensing during chemotactic migration and also in three-dimensional migration [41], [42]. Based on these observations, we silenced cofilin in HT1080 cells using siRNA and tested the cells in our invasion assay. Knockdown was confirmed by western blot and defined 48 hours post-nucleofection as the optimum time point for a 60% knockdown of the cofilin protein (Figure 5A). We observed that reduced cofilin expression failed to enhance stimulated invasion as compared to silencing HT1080 with off-target siRNA. Approximately 7% of cells treated with control siRNA invaded without mechanical stimulation, while 22% invaded when given mechanical stimulation, reflective of the enhanced invasion typically observed in untreated cells (Figure 5B). In comparison, the cofilin silenced cells showed approximately 5% invasion without mechanical stimulation and showed no significant response to the mechanical stimulation (4% invasion) (Figure 5B). Thus, while knockdown of cofilin does not impede normal invasion abilities in our assay, these results establish a role for cofilin in the enhanced invasive response invoked by mechanical stimulation.

## Discussion

The progression of cancer, from the formation and growth of the initial tumor through the multi-step metastatic cascade, is sure to be impacted by multiple mechanical factors. Within the tumor mass and in the microenvironment, factors of tissue compliance, shear force and interstitial forces are present [43]–[47]. Indeed it has been known for several years that the compliance of the tumor and its surrounding stroma are more rigid due to an enhanced deposition of ECM [13]. Matrix compliance is known to influence cell growth, morphology, differentiation and motility [48]–[52]. Changes in mechanical properties result from the unique repitoire of cells found in the tumor stroma, of most significance are the fibroblasts, myofibroblasts and pericytes [8], [9], [12]. Myofibroblasts are known to extensively remodel the ECM producing considerable forces on the deposited ECM [7], [9], [21], [53]. Pericytes associated with a tumor are different morphologically and physiologically from pericytes of normal blood vessels and forces generated by these tumor associated pericytes have been shown to alter the microvascular niche [54]–[56]. In our study we have asked whether these mechanical forces generated by remodeling and migrating cells within the stroma could impact cancer cell invasion.

The assay used for this study offers many benefits in its simplicity, yet retains some aspects of physiological relevance. For instance, the study is done in a three-dimensional environment of collagen and fibronectin, which are the most abundant ECM proteins found in the stroma of tumors, and are secreted and remodeled by cancer associated fibroblasts (CAF's) and myofibroblasts [7]. We mimic these remodeling forces, without the complication of the secreted biochemical factors that are produced by stromal cells [57]. The magnetic force generated by the paramagnetic microbeads is tuned to produce displacement forces comparable to normal fibroblasts in this culture environment (Movie S3). Furthermore, we recognized that the stellate shaped fibroblasts within the stroma typically run parallel to the basement membrane of the tumor, hence the forces applied during the remodeling are likely in this orientation, thus we applied the magnetic force in a parallel plane (see Movie S1 and S2). We also considered the range of compliance possible for a tumor and the stroma, with reports ranging from 300–2000Pa [13]. We discovered no difference in the invasive response when we tested within a range of 400–1600 Pa (Figure S2). The correct combination of these factors resulted in the enhanced invasion we were able to generate upon mechanical stimulation, however there are certain to be other factors that will further optimize this method.

Given that non-invasive cell types were unable to invade in response to the mechanical stimulation, it is reasonable to presume the necessary molecular machinery for mechanically stimulated invasion is not available. A vital structure used by highly invasive cells is the invadopodia. These structures are enriched in proteases, cytoskeletal proteins, such as actin, and adhesion proteins including α5β1 integrin [2], [58], [59]. It is likely that invadopodial structures are important in the mechanical response as they display enhanced activity to changes in compliance, which also supports our observation that the mechanically stimulated invasion is unaffected when we change the compliance (Figure S2) [1]. Cancer cell motility and invasion are actin dependent processes [38], [39], [60]. We also confirmed its requirement for mechanically enhanced invasion. Given that the response to our mechanical stimulus does not induce invasion in non-invasive cells, but enhances the existing processes, suggested a “late comer” to the established machinery (invadopodia) might participate in the mechanical sensing. Based on the fact that cofilin is not involved in the initial formation of invadopodia, but in their maturation, we evaluated it as a potential mechanical responder [40]. Our finding that knockdown of cofilin does not affect non-stimulated invasion, but eliminates the enhanced response in our assay, confirms our reasoning. What remains to be determined is if the presumed lack of maturation of the invadopodia is responsible for the loss of our response, or if there is a change in the overall number of invadopdia, or perhaps a change in the proteolytic activity of these structures.

Another intriguing observation is the requirement for fibronectin for the mechanically enhanced invasion. In our study, collagen alone did not provide sufficient signal to the cells to trigger a mechanical response. One obvious explanation is that the sensor, possibly an integrin, possessing the sensing function for enhanced invasion, does not bind to collagen, but recognizes only fibronectin as the ligand [61]. The need for fibronectin in the sensing mechanism is also consistent with several reports that mechanical load alters the structure of the fibronectin molecule, specifically the synergy site [62]–[65]. Furthermore, more recent studies find that α5β1 integrin switches fibronectin binding states based on mechanical information [18], [66], [67]. α5β1 integrin is overexpressed in a number of cancers, and is under study as both a therapeutic and diagnostic target [68]–[70]. This integrin is highly expressed at the periphery of invadopodia and is essential for the adhesion process by mediating their formation and extension [71], [72]. Our data defines significant importance to fibronectin interactions in the mechanical sensing observed in our invasion assay. We speculate the enriched expression of fibronectin receptors at the tip of invadapodia and the enhanced access granted by the pulling of the fibronectin molecules by our magnetic beads are key to this sensing mechanism, though further studies are necessary.

In conclusion, we have discovered that mechanical stimulation applied to a collagen-fibronectin matrix through micro-magnetic beads, can enhance the invasive abilities of invasive cancer cells. This response requires both extracellular and cellular proteins. From our studies we can conclusively state that the ECM component fibronectin and the cellular protein cofilin are required for this mechanical response. We further suspect invadopodia in the process of mechanically stimulated invasion. We propose these observations translate to the tumor microenvironment where multiple cell types can be found, including highly contractive cells, and that mechanical forces generated by these stromal cells could contribute to enhancing the metastatic abilities of invasion competent cells leaving the primary tumor.

## Supporting Information

Movie S1 Sequential images were captured when the magnet was absent or held stationary at various positions, demonstrating the paramagnetic bead movement upon stimulation. Bead displacements were observed to range from 0.5–5 µm under a 40X objective. Upon removal of the magnet, beads return to their original position. (1 pixel  = 0.4 µm)
(WMV)

Movie S2 Bead displacement images were taken while the magnet was rotated at 8 rpm (0.13 Hz), 1.5 cm in a 2 cm orbit above the matrix. Ten images were captured every ten seconds under 60X magnification. (1 pixel  = 0.27 µm)
(WMV)

Movie S3 Bead displacement mediated by MEF cells embedded in the matrices protruding and retracting extensions. 30 images were captured every two minutes under 40X magnification. Bead displacements are seen at different positions around the moving cell. (1 pixel  = 0.4 µm)
(WMV)

Figure S1 **Secretion of fibronectin from HT1080 cells is undetectable in collagen-only matrices.** **A)** Western blot of fibronectin from total protein extracts of HT1080 and MEF cells, cultured on standard polystyrene dishes, demonstrates reduced amounts of fibronectin from HT1080 cells. **B)** Western blot of fibronectin from collagenase treated collagen-only matrices in which HT1080 or MEF cells were cultured and stimulated for 24 hours.
(TIF)

Figure S2 **Mechanically stimulated invasion is unaffected by collagen concentrations and changes in compliance.** Invasion assays of HT1080 cells in collagen/fibronectin matrices under stimulated and unstimulated conditions. Collagen concentrations of 2.5 mg/ml (∼400 Pa) and 4.5 mg/ml (∼1600 Pa) were used; both produced similar extents of invasion (23.6% and 26.6% respectively. Data represents 3 independent experiments.
(TIF)
