# Supplementary figures and images for: Cancer Cell Invasion Is Enhanced by Applied Mechanical Stimulation
Source: PLoS One. 2011 Feb 17;6(2):e17277. doi: 10.1371/journal.pone.0017277 (PMC3040771; doi:10.1371/journal.pone.0017277)

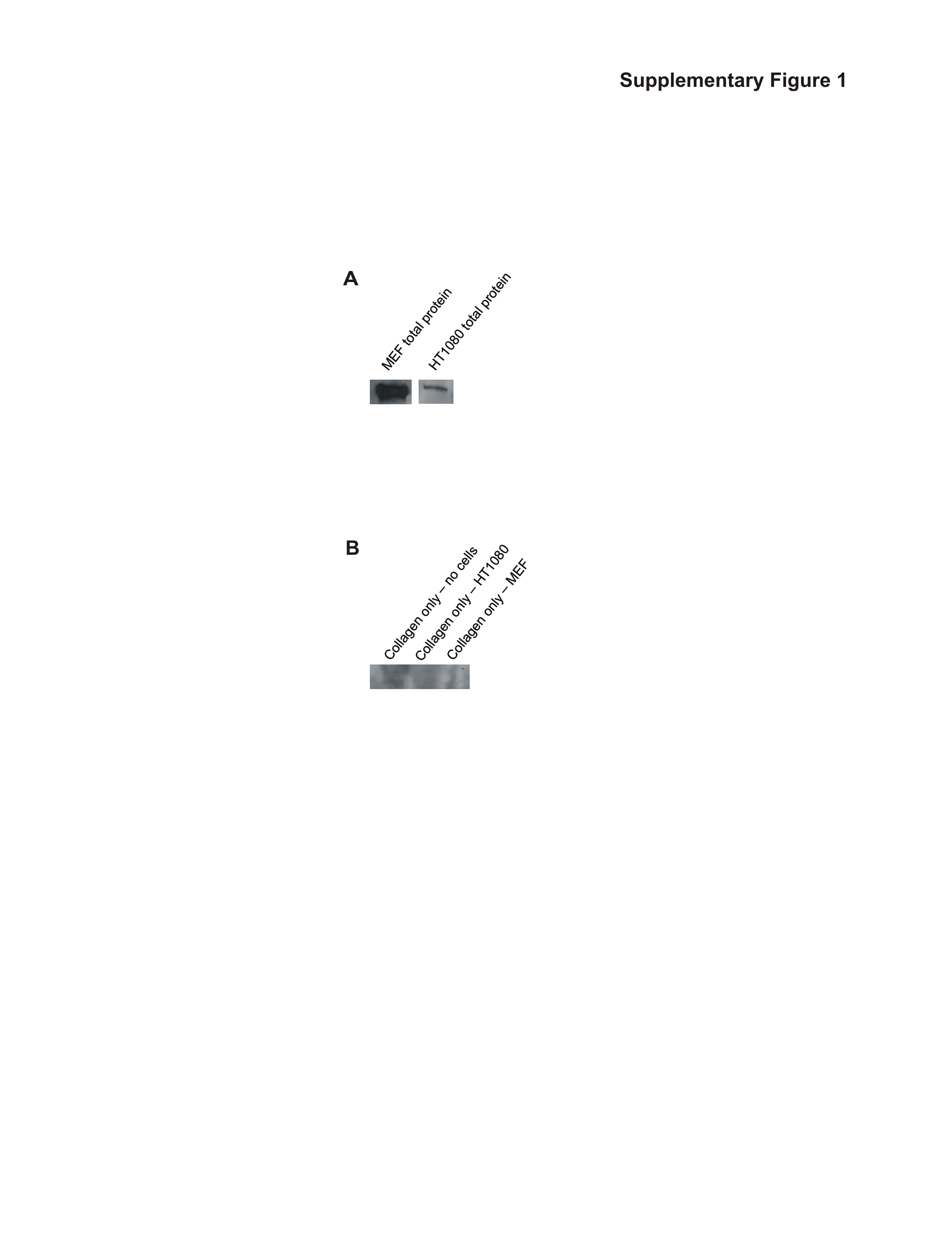

Supplement: Figure S1 — Secretion of fibronectin from HT1080 cells is undetectable in collagen-only matrices. A) Western blot of fibronectin from total protein extracts of HT1080 and MEF cells, cultured on standard polystyrene dishes, demonstrates reduced amounts of fibronectin from HT1080 cells. B) Western blot of fibronectin from collagenase treated collagen-only matrices in which HT1080 or MEF cells were cultured and stimulated for 24 hours. (TIF) [file pone.0017277.s004.tif]

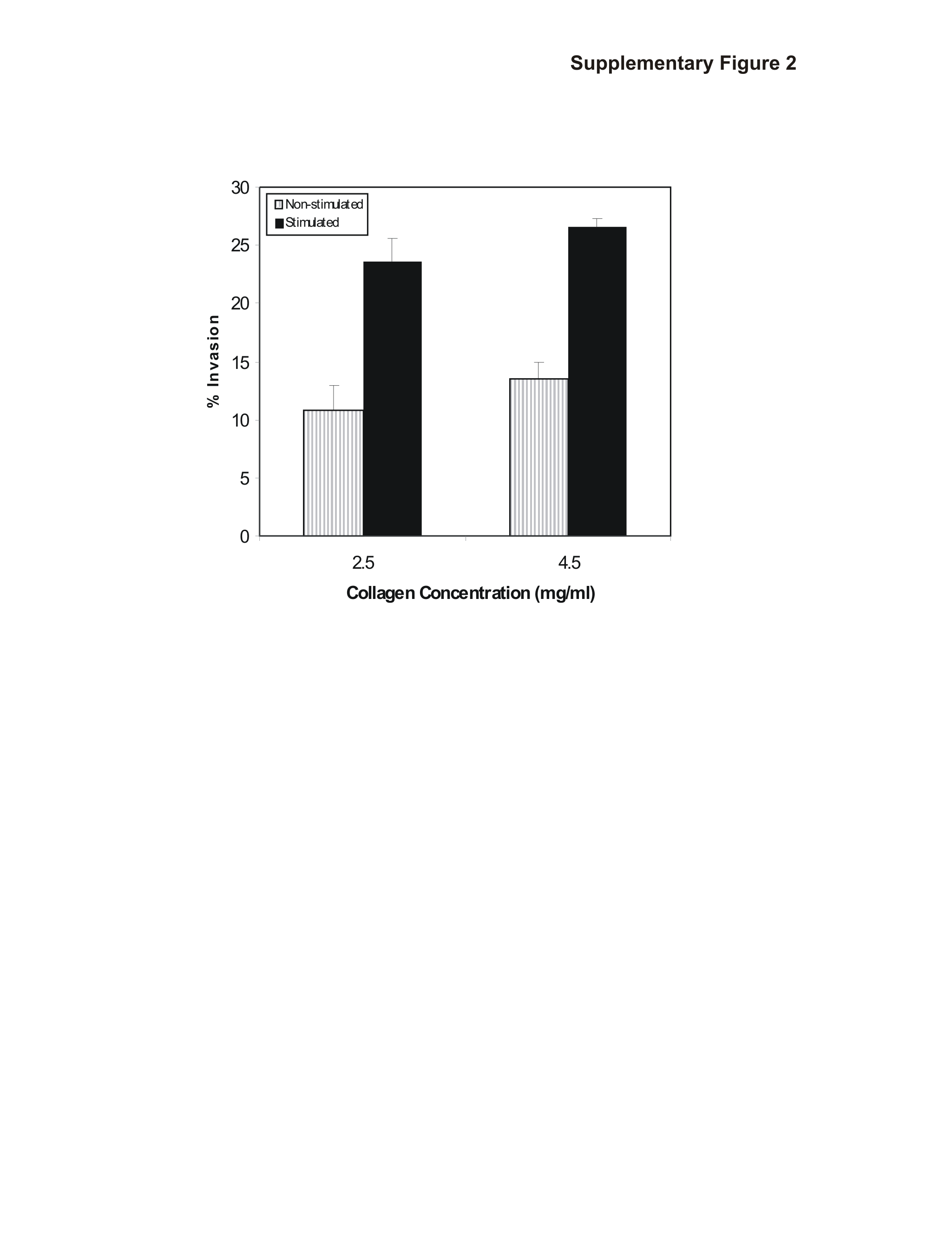

Supplement: Figure S2 — Mechanically stimulated invasion is unaffected by collagen concentrations and changes in compliance. Invasion assays of HT1080 cells in collagen/fibronectin matrices under stimulated and unstimulated conditions. Collagen concentrations of 2.5 mg/ml (∼400 Pa) and 4.5 mg/ml (∼1600 Pa) were used; both produced similar extents of invasion (23.6% and 26.6% respectively. Data represents 3 independent experiments. (TIF) [file pone.0017277.s005.tif]
